# CT and MRI imaging in Sweden: retrospective appropriateness analysis of large referral samples

**DOI:** 10.1186/s13244-023-01483-w

**Published:** 2023-08-02

**Authors:** Henriettæ Ståhlbrandt, Ida Björnfot, Torsten Cederlund, Anja Almén

**Affiliations:** 1 Department of Radiology, Hospital of the Highlands, 575 81 Eksjö, Sweden; 2 Department of Radiology, Länssjukhuset Ryhov, 551 85 Jönköping, Sweden; 3 Department for Authorisation of Radiation Applications, Swedish Radiation Safety Authority, 171 16 Stockholm, Sweden; 4 Department for Radiation Protection and Environmental Assessment, Swedish Radiation Safety Authority, 171 16 Stockholm, Sweden; 5 Medical Radiation Physics, Department of Translational Medicine, Malmö, Lund University, 205 02 Malmö, Sweden

**Keywords:** Justification, Appropriateness, Computed tomography, Magnetic resonance imaging, Referral

## Abstract

**Objectives:**

The numbers of computed tomography (CT) and magnetic resonance imaging (MRI) examinations per capita continue to increase in Sweden and in other parts of Europe. The appropriateness of CT and MRI examinations was audited using established European appropriateness criteria. Alternative modalities were also explored. The results were compared with those of a previous study performed in Sweden.

**Methods:**

A semi-automatic retrospective evaluation of referrals from examinations performed in four healthcare regions using the European appropriateness criteria in ESR iGuide was undertaken. The clinical indications from a total of 13,075 referrals were assessed against these criteria. The ESR iGuide was used to identify alternative modalities resulting in a higher degree of appropriateness. A qualitative comparison with re-evaluated results from the previous study was made.

**Results:**

The appropriateness was higher for MRI examinations than for CT examinations with procedures classed as usually appropriate for 76% and 63% of the examinations, respectively. The degree of appropriateness for CT was higher for referrals from hospitals compared to those from primary care centres. The opposite was found for MRI examinations. The alternative modalities that would result in higher appropriateness included all main imaging modalities. The result for CT did not show improvement compared with the former study.

**Conclusions:**

A high proportion of both CT and MRI examinations were inappropriate. The study indicates that 37% of CT examinations and 24% of MRI examinations were inappropriate and that the appropriateness for CT has not improved in the last 15 years.

**Critical relevance statement:**

A high proportion of CT and MRI examinations in this retrospective study using evidence-based referral guidelines were inappropriate.

**Key points:**

∙ A high proportion of CT and MRI examinations were inappropriate.

∙ The CT referrals from general practitioners were less appropriate that those from hospital specialists.

∙ The MRI referrals from hospital specialists were less appropriate that those from general practitioners.

∙ Adherence to radiological appropriateness guidelines may improve the appropriateness of conducted examinations.

**Graphical abstract:**

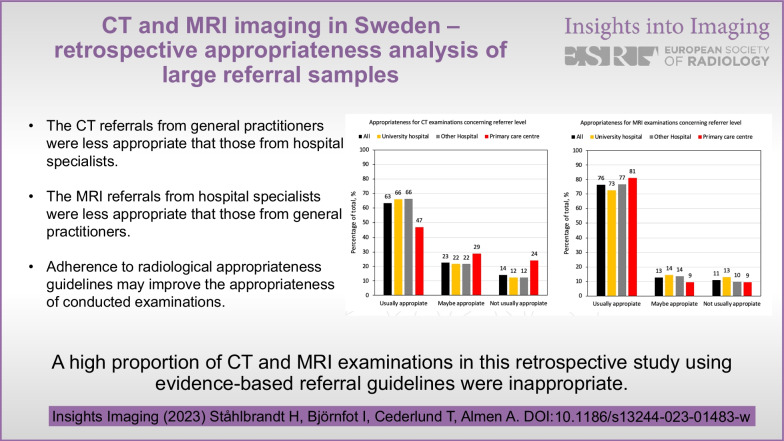

## Introduction

The use of medical imaging has increased over the past decades [1]. In particular, the numbers of CT examinations and MRI examinations have increased in frequency. The number of CT examinations carried out in Sweden increased by 130% from the year 2005 to 2018 and the number of MRI examinations increased by 104% in the same period [2]. Similar trends are observed, e.g. in Switzerland where the CT examination rate between 2013 and 2018 increased by approximately 15% and in Finland where an 82% increase between 2008 and 2018 was observed [3, 4]. In the USA, which has a higher number per capita compared to Europe, the increase in CT examinations was 20% between 2006 and 2016 [5], a smaller increase compared to Europe. There are several reasons not to carry out examinations that do not contribute to the care of the patient. One concern, regarding CT, is the radiation exposure of the patients. Today radiation dose from CT examinations constitutes a large proportion of the total radiation dose from radiological diagnostic examinations [1–4]. This may not be an issue if the output of the imaging procedure benefits the diagnosis and care of the patient in the clinical setting. However, some studies have shown that this is not always the case. National studies carried out in Finland, Sweden, Spain, Italy, Luxembourg and Ireland [6–12] indicate that 5–20% may not be justified.

The concept of justification of ionising radiation used in medical exposure, as defined in the system of radiation protection [13], applies to three levels depending on each other. The principle is complex and includes many factors other than those directly relating to the patient, e.g. economical and societal factors. The first level postulates that radiological medical examinations of patients generally are justified, and is taken as a given. The second relates to examinations with a specific objective, performed with a certain type of equipment, and assumes that there is evidence that the benefit of a specific examination exceeds the expected detriments, including radiation risks, for a specific medical indication. The third level concerns justification of using ionisation radiation in the examination of the individual patient. This requirement should be evidence based, where only an examination appropriate to the clinical indication of the patient is used. Only in exceptional cases would it be justified to expose an individual patient to ionising radiation under other conditions.

An assessment of justification, as defined in the radiation protection system, is difficult to perform. Such an assessment often has to include several aspects of the patient's care beyond the information readily available and should preferably be quantitative. However, studies to be carried out in the clinic must use a method that is not too complex, require only reasonable resources and furthermore be based largely on evidence-based criteria. Appropriateness criteria are derived to ensure the appropriate utilisation of examinations. Appropriateness criteria reflect medical necessity and the diagnostic value of examinations and have been developed with a focus on balancing possible benefit against possible detriment [14]. Appropriateness could therefore be used as a proxy for justification. That is, evaluating appropriateness is an indication of the level of justification. A novel methodology using appropriateness criteria for retrospective evaluation of examinations has been developed [15]. This study showed that it was possible to evaluate a large number of examinations retrospectively in a semi-automatic way using the database included in ESR iGuide, a clinical decision support system, as a basis for the evaluation.

The aims of this study were to (i) apply a novel method for retrospective evaluation of CT and MRI examinations, (ii) evaluate the appropriateness of CT and MRI studies performed in four regions in Sweden, (iii) explore which alternative modalities would improve appropriateness and (iv) compare the results with a previous study on CT examinations performed in 2006.

## Materials and methods

### The data set

The study included data from 13,075 completed CT examinations and MRIs of adults performed in October 2021 in four of 21 healthcare regions in Sweden and was based on extracted information in the referrals. The data set generated included data from those referrals where the clinical indication matched a clinical indication in ESR iGuide. No other specific inclusion criteria, except not including CT examination used for radiation therapy planning, were applied. The data set thus includes all types of indications such as diagnostics, treatment preparation and follow-up as well as referrals from the emergency department. The majority of examinations (78%) were CT examinations. This reflects well the distribution between CT examinations and MRI in Sweden [2]. Note that the data were collected from the radiology department that performed the examinations.

### The method of evaluation

As mentioned in the introduction, the method uses the ESR iGuide as a standard. This is a clinical decision support system, which contains a database of over 4000 clinical scenarios of which approximately 2300 are scored, i.e. gives a scored indication result, and thus provides diagnostic imaging recommendations based on peer-reviewed articles and expert panel recommendations. ESR iGuide is provided by the European Society of Radiology in close collaboration with the ACR Select provided by the American College of Radiology [14]. The evaluation method [15] was largely automated and used data in the ESR iGuide and data extracted from the referrals. The method could briefly be summarised as follows. The clinical indications, as free text, along with age and gender, were extracted from the referrals. These data were compared to the clinical indications available in the ESR iGuide using a data search engine. A data analysis tool was created to run the samples and get a scoring, regarding the examination performed, indicating the appropriateness level for each referral. Using the database in ESR iGuide, it was also possible to evaluate whether a more appropriate examination existed and which modality it comprised. Comparisons were made of the appropriateness of CT examination and MRI for all examinations overall and separately for each healthcare region. The influence of referrer affiliation on the appropriateness level for CT and MRI examinations, respectively, was also evaluated as was appropriateness by age groups. The appropriateness is categorised as (i) usually, (ii) maybe and (iii) usually not appropriate using the ESR iGuide approach.

In addition, the modalities that would have been used if the examination had been placed in a group with a higher level of appropriateness were identified. This indicated whether a different modality was more appropriate or whether the modality used would still be applied, but with a different method. This was evaluated for both CT and MRI examinations, and the respective distribution of alternative modalities was derived.

### The comparison with a previous national study

The result was compared with a previous study performed in Sweden [8]. This study included CT examinations and comprised in total of 2435 CT examinations. To compare the outcome with the present study, a re-calculation of the original study was performed. Firstly, the evaluation scale “CT was the proper modality”, “another modality should have been used” and “the examination should not be carried out at all” was used to rank the examinations. Secondly, the results from the whole country were used to compare the results of the present study due to the rather low number of examinations included. Thirdly, the referrer affiliation was re-divided into three groups: university hospitals, other hospitals and primary care centres to match the groups in the present study. In the former study, the group “other hospital” comprised two levels.

The designation “CT was the proper modality” was judged to constitute high appropriateness, i.e. to be compared with “usually appropriate” in the present study. The “another modality should have been used” and “the examination should not be carried out at all” designations were compared with “maybe” and “usually not appropriate”. A quantitative comparison is hard to perform due to the differences in methods. A qualitative comparison was therefore made regarding the overall level of appropriateness and the influence of referrer affiliation on the level of appropriateness.

## Results

### The referrals

The demographics of the data set, including data from the previous study, are given in Table 1. The regions contributed different numbers of referrals. Region D contributed approximately 40% of the total number of referrals. Region C contributed the least with around 17% of the total and the other two regions with just around 21% each. This distribution roughly applies to both CT and MRI examinations. The average subject age is similar in all regions with a typical age of 69 years. Compared to the study in 2006, the patients are somewhat older. This age difference reflects the current use of radiology, where a shift towards older patients is seen.

**Table 1:** Summary of the examinations included in the study

	Total	Region A	Region B	Region C	Region D
All examinations CT and MR	13,075	3067	2684	2156	5168
CT	10,141	2367	2090	1764	3920
MR	2934	700	594	392	1248
Age median	69	69	69	69	68
[Q1–Q3]	(52–78)	(54–78)	(51–79)	(53–79)	(51–78)
CT (2006 study)	2 435	135	192	584	228
Age median	64	68	67	64	64
[Q1–Q3] (2006 study)	(50–77)	(46–82)	(46–77)	(46–77)	(55–74)

A slight majority of referrals came from the referrer affiliation denoted as “other hospitals” (Table 2). In the 2006 study, a slightly larger proportion came from primary care centres and a slightly smaller proportion from university hospitals compared with the present study. The referrals distributed by affiliation were somewhat different in the different regions. For CT examinations, about half of the referrals came from the group called “other hospitals”. Worth noting is that for regions A and C, a small number of referrals from university hospitals are included. In this regard, regions B and D are more representative in terms of referral profile. In the case of MRI, the referral affiliation is more evenly distributed. Referrals from primary care centres constitute on average 28% of all referrals, while in the regions this figure varies between 22 and 34%.

**Table 2:** Percentage (%) of examination with reference to referrer affiliation

	Total	Region A	Region B	Region C	Region D	CT (2006)
*CT examinations*						
University hospital	34	3	69	10	45	27
Other hospitals	52	82	18	78	40	53
Primary care centre	14	15	13	12	15	20
*MRI examinations*						
University hospital	37	1	68	6	54	
Other hospitals	35	65	7	72	19	
Primary care centre	28	34	25	22	27	

### The level of appropriateness

Table 3 presents the result of appropriateness together with applied age groups. The appropriateness for MRI is higher, regardless of age group. The percentage of “usually appropriate” was 63% and 76% for CT and MRI examinations, respectively. There are no clear trends, but it seems that the values are highest for the oldest age groups. For the lowest age group, the percentage “not usually appropriate” for CT examinations is 9% compared with the average number of 14% for all ages. For MRI, the corresponding values are 15% and 11%.

**Table 3:** The level of appropriateness (%) for three score groups for all examinations and for applied age groups

Age group (y)	All	18–29	30–39	40–49	50–59	60–69	70–79	80–89
*MRI examinations*								
Usually	76	74	74	76	74	77	79	82
Maybe	13	11	13	12	14	14	12	12
Not usually	11	15	13	12	12	9	9	6
*CT examinations*								
Usually	63	71	62	61	64	60	61	67
Maybe	23	20	23	22	20	25	24	22
Not usually	14	9	15	17	16	15	15	11
*CT examinations 2006*								
CT correct modality	81	74	69	79	81	82	83	86
CT not correct modality	13	14	25	14	14	13	11	8
No exam indicated	6	12	7	6	5	6	6	5

**Fig. 1:**
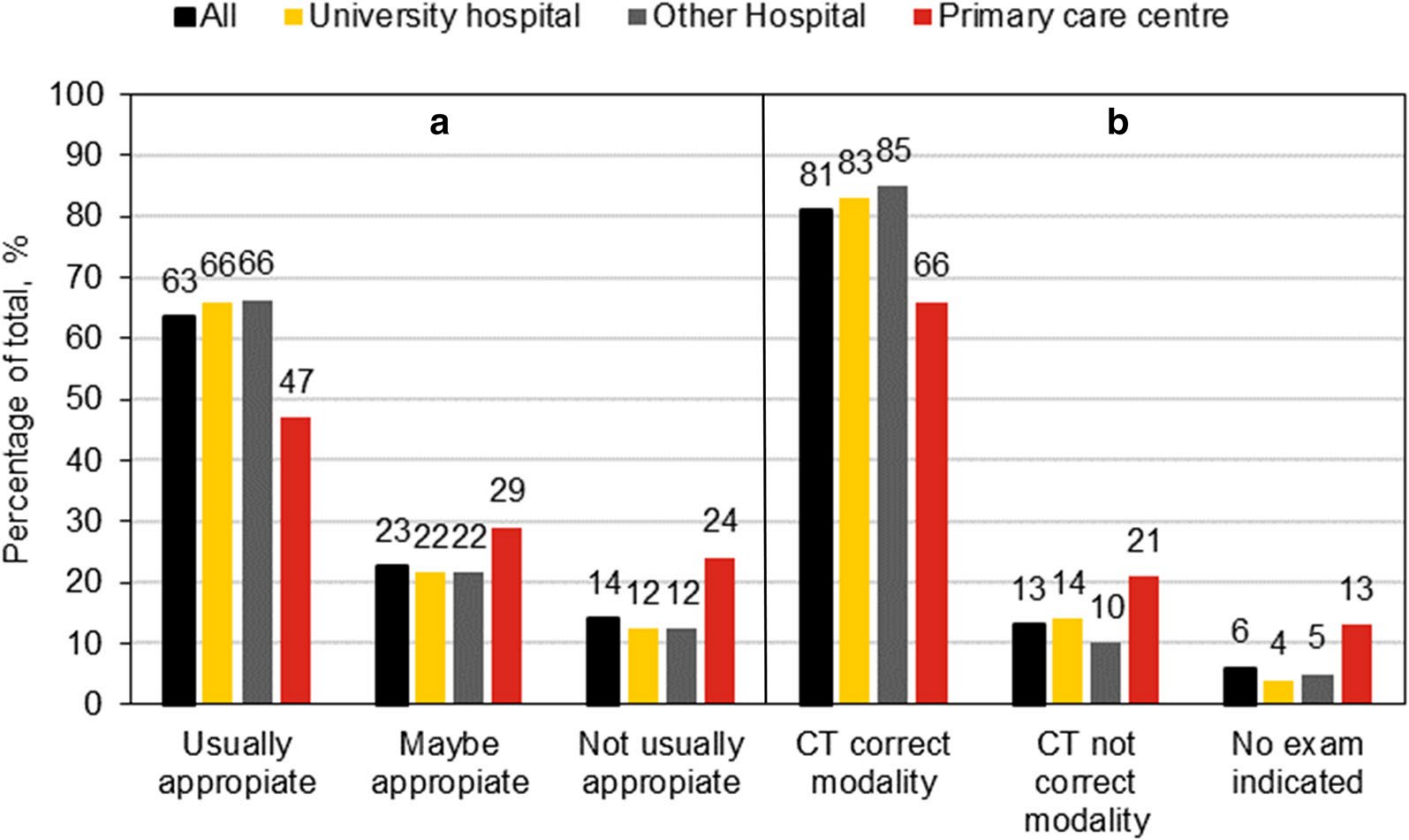
Appropriateness for CT examinations concerning referrer level. **a** The present study, **b** the 2006 study

**Fig. 2:**
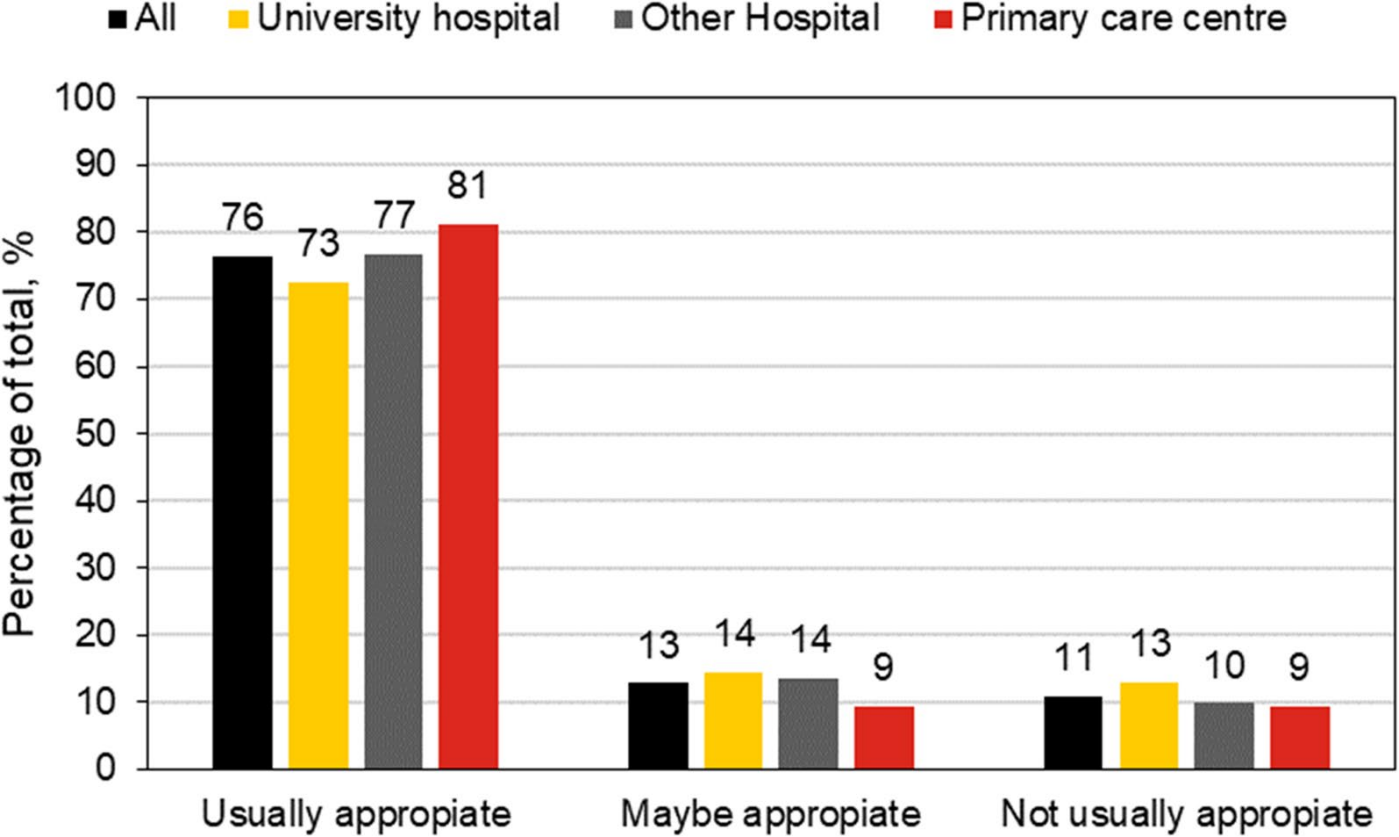
Appropriateness for MRI examinations concerning referrer level

A comparison between healthcare regions is shown in Table 4. All regions have higher appropriateness for MRI compared to CT examinations. Region B has higher levels of appropriateness for both CT and MRI compared to the other regions. However, region D is nearly as high for CT examinations. In Figs. 1 and 2, the result for different referrer levels is shown. The referrals from primary care centres scored particularly high for MRI, but low for CT examinations.

**Table 4:** The level of appropriateness (%) for three score groups for the healthcare regions A-D

	Region A	Region B	Region C	Region D	Total
*MRI examinations*					
Usually	77	80	77	74	76
Maybe	15	12	12	13	13
Not usually	8	8	11	14	11
*CT examinations*					
Usually	59	70	59	65	63
Maybe	23	20	25	22	23
Not usually	18	10	17	13	14

The total score is included for comparison

**Fig. 3:**
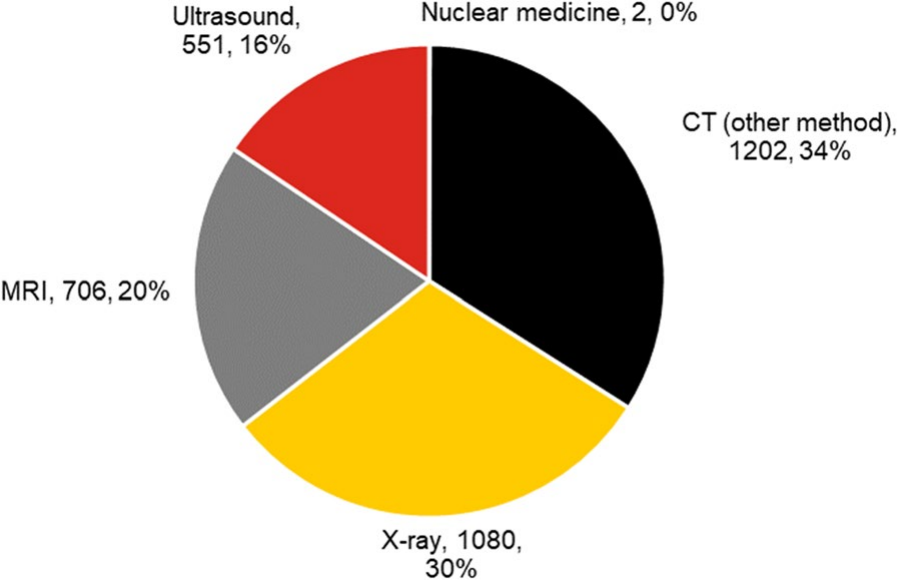
The more appropriate modality when the CT examination was scored maybe/usually not appropriate (number and percentage)

For CT examinations, 3541 examinations (35% of the total) that were designated as “maybe” or “usually not appropriate” had alternative examinations with higher appropriateness. In 2339 of these examinations (23% of the total), this involved examinations using alternative modalities and for 1202 examinations (12% of the total) different CT methods should have been applied. The modality designated as more appropriate is shown in Fig. 3. Note that a considerable percentage of examinations were conventional X-ray. No more appropriate examinations were available for 167 procedures (1.6% of the total).

**Fig. 4:**
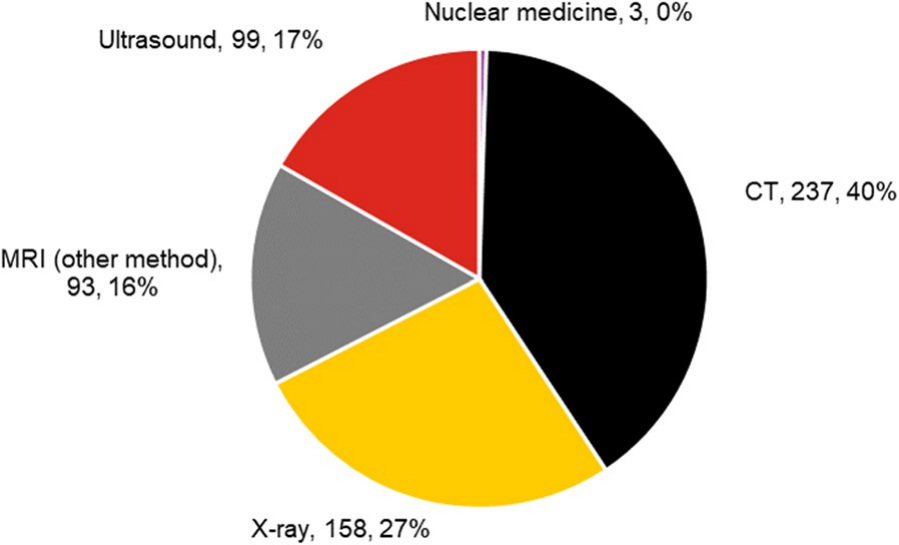
The more appropriate modality when the MRI examination was scored as maybe/not usually appropriate (number and percentages)

For MRI, 590 examinations (20% of the total) that were included in groups designated as “maybe” and “usually not” appropriate had alternative examinations with higher appropriateness. In 93 of these examinations (3% of the total), the more appropriate examination would have required an alternative MRI method. In 497 examinations (17% of the total), more appropriate studies required a change of modality as shown in Fig. 4. A considerable percentage of examinations were CT examinations. No more appropriate examinations were available for 104 examinations (3.4% of the total).

### The result compared with CT study performed in 2006

In the former study, 81% of the CT examinations were categorised as “correct modality applied”, 13% of the examinations as “another modality should have been used” and 6% as “no radiological indication was indicated” (Fig. 1). In the present study, 63% were categorised as “examination was usually appropriate”. The examinations for which a more appropriate examination could have been performed still could result in a CT examination, i.e. indicating that the correct modality was used, constituted 12%. That is, the present study indicates that for 75% of the CT examinations, the correct modality was used. That is somewhat lower than the previous study. On the lower end, in the former study, 6% of the examinations should not have been performed and in the present study, 14% were usually not appropriate. Qualitative analysis suggests that the percentage of appropriate CT examinations has not improved and it is plausible that the proportion of appropriate examinations has decreased in the last 15 years. The lower number of appropriate CT examinations for referrals from primary care centres was evident in both studies (Fig. 1), while the relative appropriateness for the younger age groups seems to be worse in the former study (Table 3).

## Discussion

In this study, the appropriateness of performed CT and MRI examinations using European criteria and a semi-automatic method was derived. The CT and MRI examinations were considered “usually appropriate” in 63% and 76% of the examinations, respectively. In most of the examinations that were considered less appropriate, other modalities or methods could be chosen to improve appropriateness. The present study suggests that there is a need for better clinical practices to improve appropriateness. Such improvement also seems justified and necessary in other European countries [16]. In previous studies, the percentage of appropriate CT examinations varied from 52 to 80% [8–11] and appropriate MRI examinations around 79% [9, 10]. The results in the present study are at similar levels, but it is difficult to make detailed comparisons because the method used varied in each study. In the future, a common methodology is warranted.

The higher appropriateness for MRI examinations may be due to the lack of MRI equipment compared to CT equipment and consequently a greater need to select the most appropriate MRI examinations to ensure correct use of a scarce resource. In many hospitals, radiographers and general radiologists assess the appropriateness of CT examinations, while MRI specialists mainly assess MRI examinations. This may also influence the differences between CT and MRI.

The comparisons between the previous Swedish study and the present study may indicate a trend towards a smaller percentage of appropriate examinations, but differences in methodology might account for this difference. This is plausible given the doubling of the number of examinations performed. Furthermore, technologies and methods in CT and MRI advance gradually and one can assume that it will become increasingly difficult for both referrers and radiologists to choose an appropriate examination without readily available guidelines. Subspecialisation may lead to better appropriateness of examinations performed. The current study does not provide a clear indication of this, as the level of appropriate examinations from university hospitals referrers is about the same for CT examinations or lower for MR examinations for referrers at other types of hospitals. For CT examinations, referrals from primary care centres have approximately twice the proportion of “generally not appropriate” examinations compared to referrals from hospitals. However, for MRI examinations, the highest percentage of appropriate examinations are referrals from primary care providers. This may be because there are more guidelines for MRI examinations at the primary care level compared with CT examinations. It could be valuable to study referrals from primary care centres in more detail.

For CT examinations, the highest proportion of suitable examinations was found in the lowest (18–29 years) and highest (80–89 years) age groups. The comparison with the previous study suggests that the relative appropriateness of examinations for younger adults has improved. This may be due to more consideration being given to radiation exposure for younger patients. For MR examinations, the highest percentage of suitable examinations was also found in the highest (80–89 years) age group. This may be because only investigations that can lead to meaningful treatment for the elderly are carried out.

There are regional differences. It is beyond the scope of this study to look at possible population differences, but national guidelines exist supporting the appropriateness of radiological examinations. Even though efforts are made to increase the number of national guidelines, care must also be taken to make available and implement these guidelines so that referrers and radiologists can easily apply them at the point of appropriateness evaluation.

The major strengths of the study are the unbiased use of appropriateness criteria and the large number of examinations included (13,075 CT and MRI examinations). Prior studies have evaluated significantly smaller samples of between 350 and 1124 examinations [6, 7, 11, 12]. An additional advantage of this study is that examinations performed in preparation for radiation therapy were excluded in the mapping process. Previous studies have included these, but as they are always appropriate, they should not be within the scope of this type of study which is focussed on diagnostic imaging.

There are some limitations to the study and its methodology. Only examinations that matched ESR iGuide indications in an automated database match were included [15] and the resulting matching rate was 52%. A value higher compared to other studies [17]. Manual analyses would have improved the matching percentage but would have been significantly more time-consuming. In addition, only four of Sweden’s 21 healthcare regions were invited to participate in the study. Finally, not using the same methodology as the previous Swedish study makes a direct comparison of results difficult. The patient's medical history or other patient-specific data were not included in the automated evaluation, such information may have influenced the assessment and outcome. The urgency of the examination and whether the examinations are available at the time of request can also explain the choice of the examination. The study also did not include any evaluation of the medical information obtained and whether this information was useful and influenced the care of the patient.

Internationally, different methods have been used to study appropriateness, which comparing studies from other countries less meaningful. It may be warranted, at least nationally, to agree on methodology to be used in clinical audits of appropriateness. Evaluation tends to be time-consuming when human observers are required and automating processes to the greatest extent should facilitate efficiency and inclusion of greater and sufficient numbers of examinations to ensure meaningful results. The result can also be highly dependent on individual experience and expertise, and the impact of this appears to be significant. Therefore, a method should rely on evidence-based criteria. This study supports these concepts. Other studies suggest and explore new techniques, such as text analysis software and other types of automated language processing, in evaluations [18–20]. This could be a way forward in the future. The inclusion of more information and data in the process could be facilitated by using artificial intelligence as has been done in other areas of medical imaging [21].

In this study, ESR iGuide was used to evaluate the appropriateness and if a clinical decision support system is used in the clinic the evaluation can be done regularly in the clinic. As far as we know, there is only one health region in Sweden, not included in the study, which uses such a system on a smaller scale and it is not fully integrated into the clinical workflow. Decision support systems as such could improve appropriateness, but full implementation into the clinical workflow has proven to be a challenge. The system also needs to be adapted to local conditions and continuously updated.

Studying appropriateness could increase the knowledge of the extent of inappropriate examinations carried out but also whether specific types of indications or patient groups need attention, e.g. referrals from emergency departments [22]. An evaluation could also be part of clinical quality assurance in order to improve clinical routines and results.

## Conclusions

A significant number of the CT and MRI examinations included in this study were inappropriate, and for most of these, other more appropriate examination options were identified using available criteria.

For CT examinations, referrals from primary care centres have by far the lowest degree of appropriateness. In contrast, MR examinations referrals from such centres have a higher degree of appropriateness compared with referrals from hospitals. There is a need for further studies to investigate the reasons for these findings.

The comparison with a former study did not indicate an improvement but rather the opposite. However, the comparison with the former Swedish study and other European studies is difficult due to the different methods used.

More work is needed to improve the level of appropriate imaging examinations. National, standardised and structured guidelines that are easy to access and use for referrers and radiology departments provide a first step to achieving this.

## Data Availability

The authors confirm that the data supporting the findings of this study are available within the article.

## Abbreviations

CT: Computed tomography
MRI: Magnetic resonance imaging
